# Rising CO_2_ enhances hypoxia tolerance in a marine fish

**DOI:** 10.1038/s41598-019-51572-4

**Published:** 2019-10-22

**Authors:** Daniel W. Montgomery, Stephen D. Simpson, Georg H. Engelhard, Silvana N. R. Birchenough, Rod W. Wilson

**Affiliations:** 1 Bioscience Department, College of Life and Environmental Sciences, University of Exeter, Exeter, UK; 2 Centre for Environment, Fisheries & Aquaculture Science (Cefas), Pakefield Road, Lowestoft, NR33 0HT UK; 3 School of Environmental Sciences, University of East Anglia, Norwich, NR4 7TJ UK

**Keywords:** Marine biology, Respiration

## Abstract

Global environmental change is increasing hypoxia in aquatic ecosystems. During hypoxic events, bacterial respiration causes an increase in carbon dioxide (CO_2_) while oxygen (O_2_) declines. This is rarely accounted for when assessing hypoxia tolerances of aquatic organisms. We investigated the impact of environmentally realistic increases in CO_2_ on responses to hypoxia in European sea bass (*Dicentrarchus labrax*). We conducted a critical oxygen (O_2crit_) test, a common measure of hypoxia tolerance, using two treatments in which O_2_ levels were reduced with constant ambient CO_2_ levels (~530 µatm), or with reciprocal increases in CO_2_ (rising to ~2,500 µatm). We also assessed blood acid-base chemistry and haemoglobin-O_2_ binding affinity of sea bass in hypoxic conditions with ambient (~650 μatm) or raised CO_2_ (~1770 μatm) levels. Sea bass exhibited greater hypoxia tolerance (~20% reduced O_2crit_), associated with increased haemoglobin-O_2_ affinity (~32% fall in P_50_) of red blood cells, when exposed to reciprocal changes in O_2_ and CO_2_. This indicates that rising CO_2_ which accompanies environmental hypoxia facilitates increased O_2_ uptake by the blood in low O_2_ conditions, enhancing hypoxia tolerance. We recommend that when impacts of hypoxia on aquatic organisms are assessed, due consideration is given to associated environmental increases in CO_2_.

## Introduction

A lack of O_2_ is one of the greatest challenges that most life can face. In terrestrial ecosystems conditions of low O_2_ are rare. In contrast low O_2_, referred to as hypoxia, is much more common in freshwater and marine ecosystems^1–4^. Hypoxia occurs because high biological demand for O_2_ can exceed the rate of O_2_ supply to the ecosystem, leading to a reduction in environmental O_2_ levels^5,6^. However, the challenges of hypoxia are not solely a result of reduced O_2_. Organisms must also contend with simultaneous but reciprocal changes in the other respiratory gas, CO_2_.

When O_2_ decreases in aquatic systems there is a corresponding increase in CO_2_^7,8^. This is a by-product of respiration, the same process that causes depletion of O_2_. As such high CO_2_ during hypoxia is ubiquitous and unavoidable. This coupling of O_2_ and CO_2_ has been highlighted numerous times in oceanographic sciences, most recently by Robinson^5^. Yet unaccountably, despite the known link between decreasing O_2_ and increasing CO_2_ during hypoxia^8,9^, the issue of increased environmental CO_2_ during periods of low O_2_ has been relatively overlooked by biologists.

Implications of rising CO_2_ during hypoxia on aquatic organisms are particularly important to address in the face of human driven climate change. Hypoxic areas are predicted to become more common and more severe, particularly in marine systems, with the de-oxygenation of the world’s oceans recently highlighted as a major component of climate change^2,3,10–12^. In addition, there will be an increase in ambient CO_2_ as rising atmospheric CO_2_ is absorbed by the world’s oceans^13^. Non-linear interactive effects between higher atmospheric CO_2_ and CO_2_ accumulation during hypoxia will lead to increased CO_2_ levels during hypoxia in future oceans^14^. This means that effects of rising CO_2_ during hypoxia in marine systems will be amplified by climate change.

Typically, experiments which test responses to hypoxia or impacts from hypoxia on aquatic organisms create hypoxic conditions by off-gassing oxygen from water by gassing with pure nitrogen or a mix of nitrogen (N_2_) and O_2_ (for examples see^15–18^). This creates low O_2_ conditions without the concurrent CO_2_ increase that would be expected in the environment. The lack of studies in which an environmentally realistic simultaneous decrease in O_2_ and increase of CO_2_ have been conducted may lead to mismeasurement of responses to hypoxia. Recently, several studies on marine fish and invertebrates have demonstrated interactive effects of low oxygen and increased CO_2_^9,19,20^, with some species exhibiting loss of equilibrium (LoE) and death at higher O_2_ concentrations when CO_2_ is simultaneously elevated^19,21^. However, these experiments do not give insight into the physiological mechanisms underlying the influence of CO_2_ on hypoxic responses of fish.

Previously observed impacts of hypoxia-associated rises in CO_2_ on hypoxia tolerance of fish could be a result of changes in O_2_ uptake, as CO_2_ has been shown to impact upon several aspects of organismal biology that are involved in O_2_ uptake and transport^22,23^. We aimed to assess whether concurrent increases in CO_2_ during decreases in O_2_ affect O_2_ uptake in a marine fish, the European sea bass (*Dicentrarchus labrax*) by conducting a standard critical O_2_ level test (O_2crit_). Under normal O_2_ (normoxic) conditions fish maintain a minimum level of O_2_ consumption rate (${\dot{{\rm{M}}}{\rm{O}}}_{2}$), referred to as the standard metabolic rate (SMR), in order to meet maintenance energetic demands of essential processes through aerobic respiration^24^. As the level of O_2_ in water drops fish deploy a number of responses (i.e. increased ventilatory water flow and cardiac output, increased haematocrit, functional changes in gill morphology, changes in Hb-O_2_ affinity) in order to maintain and regulate this minimum level of ${\dot{{\rm{M}}}{\rm{O}}}_{2}$ ^25–27^. If environmental O_2_ continues to drop there comes a point at which fish are unable to regulate ${\dot{{\rm{M}}}{\rm{O}}}_{2}$ to meet minimal energy demands, referred to as the critical O_2_ level (O_2crit_). At O_2_ levels below O_2crit_ fish become oxy-conformers (where ${\dot{{\rm{M}}}{\rm{O}}}_{2}$ is directly proportional to environmental O_2_ availability) and fish become increasingly reliant on anaerobic metabolism which is unsustainable in the medium to long term. In the past the measure of O_2crit_ has been used as a proxy for overall hypoxia tolerance but recently this approach has been questioned^28,29^. Nevertheless O_2crit_ does provide information related to the ability of fish to maintain O_2_ uptake and supply during hypoxia, and its prevalence in the literature allows comparison of responses between species^30^. Furthermore, we investigated whether any changes in O_2crit_ could be linked to changes in blood acid-base chemistry and blood gas transport via alteration of Hb-O_2_ binding caused by rising environmental CO_2_. Our hypothesis was that the simultaneous increase in CO_2_ during a progressive decrease in O_2_ would decrease hypoxia tolerance (increase O_2crit_) and that this response may be a result of blood acid-base disturbance decreasing Hb-O_2_ affinity and O_2_ transport.

## Results

### O_2crit_ tests

**Figure 1:**
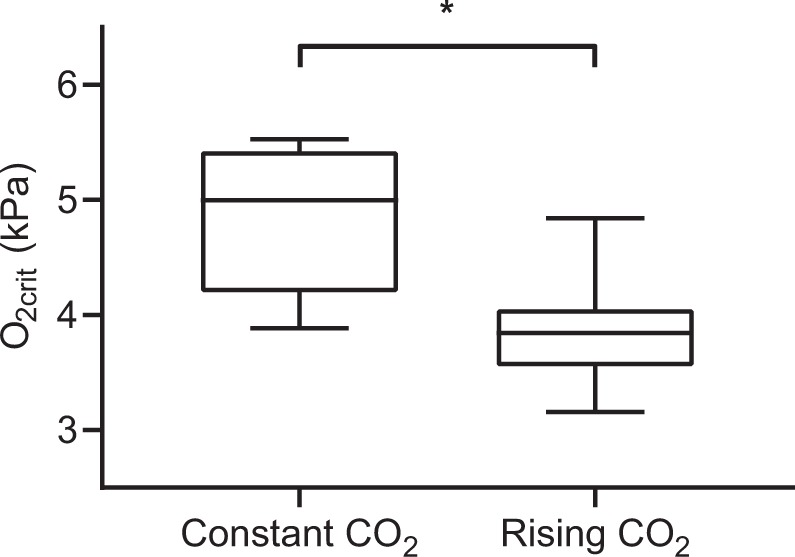
Calculated critical oxygen level (O_2crit_) of European sea bass, *Dicentrarchus labrax*, when O_2crit_ tests include a constant ambient CO_2_ level (~500 μatm, N = 8) or an ecologically realistic rise in CO_2_ (~500–2500 μatm, N = 7) during the test. *Indicates significant difference between CO_2_ regimes (p < 0.05). Boxes represent median value and inter-quartile range, whiskers represent minimum and maximum values.

There was evidence of enhanced tolerance to hypoxia in sea bass exposed to rising compared to constant CO_2_ conditions. This was indicated by measurements of O_2crit_ in European sea bass being significantly different between fish exposed to either constant, or rising CO_2_ levels during O_2crit_ tests when accounting for variation in SMR (Fig. 1; ANCOVA, F_1,12_ = 7.525, p = 0.0178). A CO_2_ increase during O_2crit_ tests resulted in a 20% reduction of O_2crit_ (3.88 ± 0.19 kPa O_2_, 18.7 ± 0.9% air saturation, mean ± S.E.) when compared to tests in which CO_2_ levels were maintained at ambient levels (4.87 ± 0.22 kPa O_2_, 23.4 ± 1.1% air saturation, mean ± S.E.).

### Blood chemistry analysis

**Figure 2:**
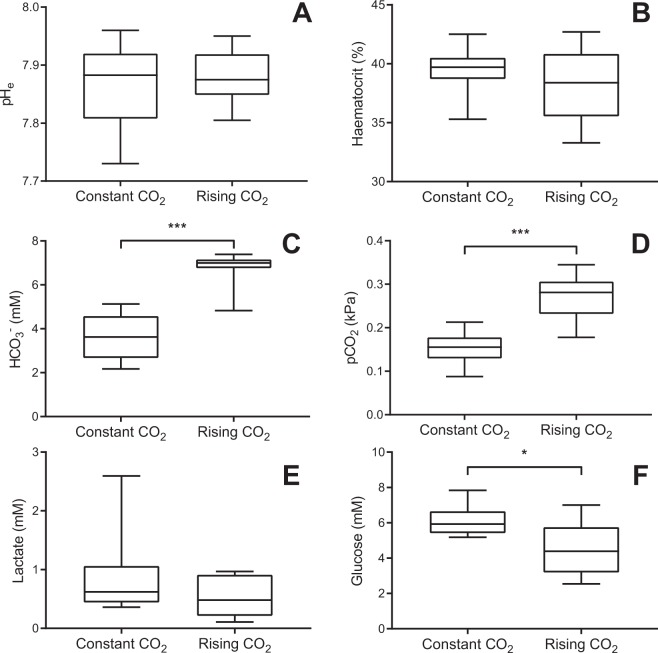
Blood chemistry characteristics of European sea bass sampled at ~8.4 kPa O_2_ (~40% air saturation) following a progressive O_2_ decline accompanied by either constant CO_2_ (~650 μatm CO_2_, N = 8) or a progressive increase in CO_2_ (sampled at ~1770 μatm CO_2_, N = 8). Blood pH (**A**), haematocrit (**B**), plasma lactate (**E**) and plasma glucose (**F**) were directly measured whilst blood *p*CO_2_ (**C**) and plasma HCO_3_^−^ (**D**) were calculated (see Methods for details). *Indicates statistical significance with p < 0.05 and ***indicates statistical significance with p < 0.001. Boxes represent median value and inter-quartile range, whiskers represent minimum and maximum values.

A comparison of blood chemistry parameters between the two treatment groups indicated that sea bass fully compensated for the rise in CO_2_ during hypoxia within 5 hours (the period of exposure prior to blood sampling). Blood pH (pH_e_) was not different between fish exposed to constant ambient CO_2_ (7.87 ± 0.03) and fish exposed to progressively rising CO_2_ (7.88 ± 0.02) (Fig. 2A, GLM, F_1,14_ = 0.23, p = 0.64). The acidifying effect of the ~79% rise in blood pCO_2_ levels in the rising CO_2_ regime (0.272 ± 0.019 kPa CO_2_) compared to the constant ambient CO_2_ regime (0.152 ± 0.015 kPa CO_2_) (Fig. 2C, GLM, F_1,13_ = 23.9, p < 0.001) was fully compensated by elevating blood HCO_3_^−^ (Fig. 2D, GLM, F_1,13_ = 40, p < 0.001). Plasma HCO_3_^−^ was 88% higher under the rising CO_2_ regime (6.76 ± 0.29 mM) when compared to the constant ambient CO_2_ (3.60 ± 0.43 mM). There were no differences in haematocrit (Fig. 2B, general linear model, F_1,13_ = 0.69, p = 0.42) or plasma lactate (Fig. 2E, general linear model, F_1,14_ = 1.48, p = 0.24) between fish sampled under a constant ambient CO_2_ regime (haematocrit = 39.4 ± 0.8%, lactate = 0.88 ± 0.26 mM) or rising CO_2_ regime (haematocrit = 38.2 ± 1.1%, lactate = 0.53 ± 0.12 mM). Blood glucose levels were ~26% lower in fish exposed to a progressively rising CO_2_ regime (4.50 ± 0.53 mM) when compared to a constant ambient CO_2_ regime (6.09 ± 0.31 mM) (Fig. 2F, GLM, F_1,14_ = 6.74, p = 0.021).

### Haemoglobin affinity for oxygen

**Figure 3:**
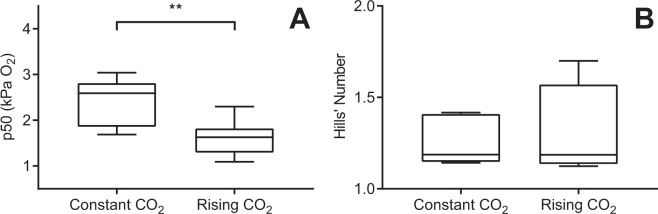
Haemoglobin P_50_ (**A**) and Hills number (**B**) for fish sampled at ~8.4 kPa O_2_ (~40% air saturation) following a progressive O_2_ decline accompanied by either constant CO_2_ (~650 μatm CO_2_, N = 7) or a progressive increase in CO_2_ (sampled at ~1770 μatm CO_2_, N = 7). Measurements were made using a gas mix which matched the calculated blood *p*CO_2_ of each individual fish blood sample. **Indicates statistical significance with p < 0.01. Boxes represent median value and inter-quartile range, whiskers represent minimum and maximum values.

Oxygen affinity of haemoglobin was increased in fish sampled under the progressively rising CO_2_ regime (Fig. 3A, GLM, F_1,12_ = 10.42, p = 0.0073). Haemoglobin P_50_ was decreased by ~32.5% in fish under the progressively rising CO_2_ regime (1.64 ± 0.15 kPa O_2_, 7.9 ± 0.7% air saturation) compared to fish sampled with a constant ambient CO_2_ (2.43 ± 0.20 kPa O_2_, 11.7 ± 1% air saturation). There was no significant change in Hills number between treatments (Fig. 3B, GLM, F_1,12_ = 0.50, p = 0.494).

## Discussion

Our results highlight the biological importance of simultaneously rising CO_2_ under conditions where O_2_ levels in water are depleted – a scenario that reflects the natural conditions during hypoxia which will be exacerbated by climate change – by demonstrating that ecologically relevant changes of CO_2_ impact physiological performance of a marine fish at both the molecular and whole organism level. We hypothesised that rising CO_2_ during progressive O_2_ decreases would lead to an increase in O_2crit_ as a result of increased blood CO_2_, decreased blood pH and the associated Bohr/Root effect of fish haemoglobin (Hb), in which Hb-O_2_ affinity (Bohr effect) and the total capacity of Hb for O_2_ (Root effect) are reduced when pH falls. In contrast, we show that increasing CO_2_ as O_2_ declined led to enhanced hypoxia tolerance of sea bass with a 20% lower critical oxygen level (Fig. 1). This change in whole organism hypoxic response was accompanied by an increase in Hb-O_2_ affinity of blood cells in fish exposed to concurrent CO_2_ rises (Fig. 3). The change in Hb-O_2_ affinity was not accompanied by a change in blood pH (although the one hour acclimation to treatment *p*CO_2_ prior to blood sampling may have contributed to this result). This provides a potential mechanistic basis to explain improved O_2crit_, enabling sea bass to enhance O_2_ uptake during hypoxia and thus maintain normal aerobic metabolism to lower environmental O_2_.

The driver of increased Hb-O_2_ affinity in sea bass exposed to concurrent O_2_ decline and CO_2_ rise is not clear from our results. Several allosteric factors that can modulate the affinity of haemoglobin for O_2_ could be involved, including pH, organic phosphates and inorganic ions. Fish haemoglobin is highly sensitive to pH, which modulates Hb-O_2_ affinity and carrying capacity via the Bohr and Root effects^31^, but we found no differences in blood pH of sea bass between treatment groups. In addition, the *in-vivo* increase in *p*CO_2_ in fish exposed to concurrent CO_2_ rises during hypoxia led to an opposite response of Hb-O_2_ affinity than would be expected by an *in-vitro* rise in *p*CO_2_ which would result in pH induced Bohr/Root effects. Increased Hb-O_2_ affinity could result from increased intracellular pH of erythrocytes^32^, as acute hypoxic exposure has been shown to stimulate a β-adrenergic stimulated increase in intracellular erythrocyte pH in rainbow trout^33^. Alternatively, increased Hb-O_2_ affinity could be due to decreased red cell nucleoside triphosphates (NTPs)^31,34^, a known hypoxia adaptation, but this can take more than 6 days to complete^35^. Sea bass may also have a particularly strong β-adrenergic response and/or fast NTP response, although there is little evidence to suggest this because P_50_ measurements from fish exposed to hypoxia at ambient CO_2_ levels do not differ from P_50_ measurements in normoxic fish from the same population (Montgomery *et al*., Unpublished data). It is possible that rising CO_2_ during hypoxia may modulate the β-adrenergic response and/or the red cell NTP response within the time frame (~4–6 hours) of our treatments. However, direct measurements of red cell pH_i_ and NTP content of sea bass in each treatment group would be needed to confirm this.

A third possible driver of increased Hb-O_2_ affinity in sea bass exposed to concurrent CO_2_ rise during hypoxia could be decreased erythrocyte chloride (Cl^−^)^36^. Although erythrocyte Cl^−^ was not directly measured in our study, plasma HCO_3_^−^ was approximately 3 mM higher in fish exposed to concurrent CO_2_ rises during hypoxia than fish which experienced constant ambient CO_2_ during hypoxia (Fig. 2D). The higher HCO_3_^−^ in fish exposed to rising ambient CO_2_ during hypoxia is likely a result of rapid compensation for a respiratory acidosis due to rising blood pCO_2_. This change in plasma HCO_3_^−^ is typically mirrored by a reciprocal change in plasma Cl^−^ ^37,38^ which is likely to be followed by a similar decline in erythrocyte Cl^−^.

Analysis of O_2crit_ is a common measure of hypoxia tolerance in fish but concurrent CO_2_ increases during hypoxia have been generally unaccounted for. A recent meta-analysis by Rogers *et al*.^39^ constructed a database of O_2crit_ research of fish (both freshwater and marine). This analysis identified two broad methods employed in O_2crit_ measurements:

1. Closed respirometry where O_2_ is usually reduced by the O_2_ consumption of the fish (52 identified studies) or;
2. Intermittent or flow-through respirometry in which O_2_ is usually reduced via gassing with pure N_2_ or combined N_2_ and O_2_ mixes (32 identified studies).

The use of closed respirometry in the majority of studies would result in concurrent CO_2_ rises as O_2_ is depleted by fish O_2_ consumption. The increase in ambient CO_2_ during closed respirometry is well known and often used as a criticism of this respirometry technique^40^. In contrast, use of intermittent-flow respirometry in O_2crit_ trials normally necessitates the reduction of O_2_ in the water by aeration with N_2_ or a mix of N_2_ & O_2_. As a result CO_2_ would likely decrease during the time course of hypoxia induction (as the gas mixture would contain zero CO_2_, rather than ~400 µatm present in atmospheric air). Such a change in CO_2_ during the O_2crit_ trial would be the opposite of that seen in nature. Therefore it may be considered that closed respirometry provides conditions which give a more environmentally relevant measure of O_2crit_^28^.

As our results indicate that rising CO_2_ during hypoxia directly affects the ability of sea bass to maintain O_2_ uptake, it could be expected that the use of closed respirometry methods would result in lower measurements of O_2crit_ than intermittent-flow methods for the same species. This effect has not been documented for species in which a direct comparison has been made – with either there being no effect of respirometry method on O_2crit_^39,41^ or higher O_2crit_ measurements when closed respirometry is used^41^. However, such comparisons are complicated by differences in the rate of hypoxia induction (RHI) by different studies, which in turn will influence how much time fish have to regulate blood pH when ambient CO_2_ is rising. For example, Regan and Richards^41^ have demonstrated that the faster rates of hypoxia induction (RHI) typical of closed respirometry O_2crit_ trials lead to higher values of O_2crit_ (i.e. lower hypoxia tolerance) when compared with longer trials using slower RHI’s typical of the intermittent-flow method. The effect of RHI on O_2crit_ was proposed by Regan and Richards^40^ as a potential explanation of the results of Snyder *et al*. (i.e. higher O_2crit_ in closed respirometry compared to intermittent-flow respirometry)^42^. The speed of RHI during closed respirometry will also effect the speed of CO_2_ rise. Almost all studies using closed respirometry to measure O_2crit_ do not report changes in CO_2_ over the course of measurement period. When accumulation of CO_2_ during a closed respirometry O_2crit_ trial was measured by Regan and Richards, CO_2_ levels were ~8,000 μatm after ~90 minutes^41^. However, we should note that this *p*CO_2_ level was measured after the O_2crit_ point, when anaerobic metabolism continues to produce CO_2_ in the absence of O_2_ consumption, but also metabolic acid production and excretion further drives up water *p*CO_2_ in the respirometer due to excess H^+^ ions titrating ambient HCO_3_^−^ to CO_2_. Regardless, at O_2_ levels above O_2crit_ the rate of CO_2_ onset will be faster than those used in our current study as a result of the faster RHI. Increased speed of CO_2_ onset in closed respirometry trials may ‘outstrip’ the ability of fish to acid-base regulate, causing an uncompensated respiratory acidosis during the time of the trial, which in turn would decrease Hb-O_2_ affinity via the Bohr & Root effects and potentially increase O_2crit_. Similarly, fish species which have reduced ability to acid-base regulate may have an increased O_2crit_ when rising CO_2_ is included in trials.

Our results indicate improved hypoxia tolerance during rising CO_2_ in European sea bass. This contrasts with previous research investigating interactive effects of CO_2_ and hypoxia on O_2crit_ of fish. Woolly sculpin, *Clinocottus analis* (an intertidal species that can breathe air), exposed to ~1100 μatm CO_2_ showed no impact on O_2crit_ after 7 days acclimation but after 28 days had O_2crit_ measurements ~34% higher than fish held in ambient (~400 μatm) conditions^43^. Higher O_2crit_ after 28 days corresponded with higher RMR and Na^+^, K^+^, ATPase activity. This contrast in results could indicate that the beneficial effect of acute rises in CO_2_ associated with natural hypoxia documented in our study are potentially reversed when fish are exposed to long term constantly high CO_2_ associated with anthropogenic climate change. Moreover, acute changes in CO_2_ had no effect on O_2crit_ of the estuarine fish species mummichog, *Fundulus heteroclitus*, and Norfolk spot, *Leiostomus xanthurus* when they were exposed to ~8,000–10,000 μatm CO_2_ immediately prior to an O_2crit_ trial^44^. As such the effect of CO_2_ on O_2crit_ will likely depend on differences in physiological responses to CO_2_ and O_2_ between species.

Simultaneously rising CO_2_ also shows variable impacts on non-metabolic responses to hypoxia of several species. Cycling CO_2_ had no effect on aquatic surface respiration (ASR), the use of the thin surface layer of water for aquatic respiration^45^, or survival in juvenile *Menidia menidia*, *Fundulus majalis*, *Fundulus heteroclitus* or *Morone saxatalis* exposed to short term cycles of O_2_^46^. In contrast, combined hypoxia and acidification resulted in an increase in the O_2_ level at which *Menidia menidia* and *Menidia beryllina* first performed ASR, consistently performed ASR, exhibited LoE, and finally died^21^. Additionally, combined high CO_2_ (~2,000 μatm) and hypoxia had no effect on survival of larval *Cyprinodon variegatus*, an additive negative effect on larval *Menidia beryllina*, and a synergistic negative effect on larval *Menidia menidia*^47^. This variation in effect of CO_2_ on hypoxia responses could be a result of methodological differences (e.g. constant high CO_2_ in Dixon *et al*.^46^, cycling DO/pH in DePasquale *et al*.^47^, and concurrent CO_2_ rise/O_2_ decrease in Miller *et al*.^21^), the level of CO_2_ used in studies (e.g. CO_2_ levels used by Miller *et al*.^21^ were ~23,000 μatm which is much higher than levels likely to be commonly found in the environment during hypoxia and may have contributed to the negative effects of rising CO_2_ noted in the study), differences in species and life stages used (changes in physiological tolerance across life stages have been noted for thermal tolerance by Komoroske *et al*.^48^), or possibly variability in response as a result of differences in previous environmental experience^49^. The role of environmental variability in species sensitivities to CO_2_ has recently been outlined in the proposed Ocean Variability Hypothesis (OVH)^50^ and warrants testing on various species in the future.

Overall our results indicate that the environmentally realistic, simultaneous rises in CO_2_ during a hypoxic event increased the hypoxia tolerance (i.e. reduces O_2crit_) of European sea bass which is at least partly explained by an enhanced ability of fish to uptake O_2_ via increased Hb-O_2_ affinity. Miller *et al*.^21^ also demonstrated impacts of concurrent CO_2_ rise on measurements of hypoxia tolerance, although in an opposite direction to that noted in our study. As concurrent CO_2_ rises during hypoxia are the norm in nature, evidence that this affects physiology of organisms exposed to hypoxia highlights an important shortcoming of research to predict tolerances to hypoxia of fish. More research on this issue is needed to clarify how common this modifying effect of CO_2_ on the response to hypoxia is and whether such measurements in the lab are ecologically relevant. A greater understanding of this issue may allow more accurate assessments of the impacts of hypoxic events on marine fish in nature, aiding management and conservation of fish species. With specific regard to measurements of O_2crit_ we believe future studies should include concurrent rising CO_2_ in the following ways:

1. Intermittent-flow respirometer studies should include increases of CO_2_ relevant to hypoxic events that organisms may experience,
2. Closed respirometry studies should report the start and end CO_2_ levels in the respirometer.

In addition, both methods should aim to create an environmentally relevant rate of hypoxia induction/CO_2_ increase for the species studied, and consistently report CO_2_ levels measured. By incorporating these recommendations we believe that future studies of O_2crit_ will give more representative estimations of species hypoxia tolerance.

## Materials and Methods

### Fish collection and husbandry

We collected juvenile sea bass from estuaries and coastal lagoons on the south Dorset coast and Isle of Wight in June 2017 (Marine Management Organisation permit #030/17 & Natural England permit #OLD1002654). Prior to experimentation, these fish were held in the University of Exeter’s Aquatic Resource Centre in an aerated recirculating aquaculture system and fed a commercial pellet at a ration of ~1–2% body weight per day three times a week (for system water chemistry see Table 1). All fish were starved for a minimum of 72 hours prior to the start of all measurements to ensure their metabolism was not affected by digestion (i.e. specific dynamic action^24^). All experimental procedures were carried out under home office licence P88687E07 and approved by the University of Exeter’s Animal Welfare and Ethical Review Board.

**Table 1:** Water chemistry parameters of the recirculating aquaculture system in which sea bass were held prior to experimental work (means ± S.E. shown).

Time held in system (days)	Temperature (°C)	pH	Salinity	Total Alkalinity (mM/kgSW)	*p*CO_2_ (μatm)
318	18.01 ± 0.03	8.04 ± 0.04	33.25 ± 0.70	2064.6 ± 136.0	516.9 ± 41.1

### Measuring hypoxia tolerance

We determined oxygen consumption rates (${\dot{{\rm{M}}}{\rm{O}}}_{2}$) of sea bass using an intermittent-flow respirometer system. The respirometer system set up followed recommendations set out by Svendsen *et al*.^51^. Briefly, the system comprised of a sealed 4.515 L respirometer chamber connected to a recirculating loop, including an in-line recirculating pump (Eheim universal 600, Deizisau, Germany), and a measurement chamber into which a temperature-compensated fibre optic oxygen optode (Firesting O_2_ oxygen meter, Pyroscience GmBH, Germany) was placed. Oxygen optodes were calibrated in water at the start of experiments at 100% air saturation and 0% air saturation according to manual instructions. Respirometry was conducted in a semi-closed system consisting of three 100 L experimental tanks fed by a 100 L sump, with overflowing water from the experimental tanks recirculating back to the sump. A second pump was used to periodically flush the respirometer system with water from the surrounding tank. This pump was controlled by an automated computer program (AquaResp 3, AquaResp®) to intermittently flush the respirometer. Five respirometer chambers were distributed between the three experimental tanks (maximum of 2 chambers per experimental 100 L tank). The sump was temperature controlled (18.27 ± 0.02 °C, mean ± S.E.) using a heater/chiller unit (Grant TX150 R2, Grant Instruments, Cambridge, UK) attached to a temperature exchange coil. Together these tanks formed a 400 L system with the same temperature, oxygen and water chemistry parameters for all respirometers.

Individual sea bass (average mass = 131.2 ± 7.5 g), chosen at random, were placed inside the respirometers and allowed an overnight recovery period, for a minimum of 13 hours, before O_2crit_ tests began. While sea bass were in the respirometers measurements of ${\dot{{\rm{M}}}{\rm{O}}}_{2}$ were conducted every 10 minutes, including a flush period of 300 s, a wait period of 60 s and a measurement period of 240 s. During the wait and measurement period the chamber was sealed by switching off the flush pump and the decline in dissolved O_2_ within the chamber was continuously measured by the fibre optic O_2_ electrode.

Following the overnight recovery period a standard O_2crit_ test was conducted. Oxygen levels in the respirometer system were reduced from ~100% air saturation to ~15% air saturation over the course of 6 hours (decline in O_2_ was ~20% air saturation per hour between ~100% air saturation and ~40% air saturation and ~10% air saturation per hour from ~40% air saturation until the end of trials). Oxygen was regulated by gassing the sump and experimental tanks with a mix of N_2_ and O_2_ (G400 Gas mixing system, Qubit Biology Inc.) at a rate of 10 L min^−1^ following a pre-set automated protocol (Flowvision, Alicat software). Levels of CO_2_ within the system were controlled under one of two treatments (with 8 fish exposed to Treatment 1 and 8 separate fish exposed Treatment 2):

- Treatment 1 (Constant CO_2_) - ambient levels of CO_2_ were maintained by including 0.04% CO_2_ as part of the gas mix delivered to the respirometer system.
- Treatment 2 (Rising CO_2_) - the proportion of CO_2_ in the gas mix was gradually increased as O_2_ was decreased. This increase in CO_2_ was designed to reflect environmentally realistic increases in CO_2_ predicted as a result of depletion of O_2_ by bacterial respiration (assumed respiratory quotient of 1)^52^, using the seawater carbonate chemistry calculator CO2sys (see supplementary material for predictions of increased CO_2_ during hypoxia).

**Figure 4:**
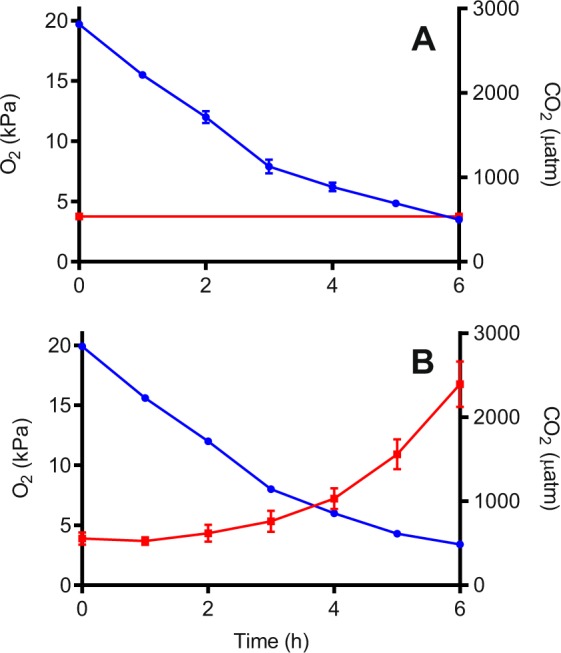
Changes in partial pressure of O_2_ (expressed as kPa O_2_) and CO_2_ (μatm) during (**A**) two O_2crit_ trials representing treatment 1 in which O_2_ was reduced with no change in CO_2_; and (**B**) two trials representing treatment 2 where O_2_ was reduced with a corresponding rise in CO_2_. Data presented are means ± S.D.

Water chemistry of treatment 2 was monitored once per hour by measuring pH_NBS_, temperature and salinity as well as taking a 12 mL water sample to measure total CO_2_ (TCO_2_)/Dissolved Inorganic Carbon (DIC). Water chemistry of treatment 1 was monitored at the start and end of the treatment to ensure no change in water *p*CO_2_ occurred over the time course of the O_2crit_ trial. Seawater DIC analysis was conducted using a custom built system described in detail by Lewis *et al*.^53^. These four parameters were then input into the seawater carbon calculator programme, CO2SYS to calculate *p*CO_2_ based on the NBS pH scale, equilibration constants from Mehrbach *et al*. refitted by Dickson and Millero, and KSO_4_ dissociation constants from Dickson. The reciprocal changes in O_2_ and CO_2_ during O_2crit_ tests for each treatment are illustrated in Fig. 4. O_2crit_ tests were stopped once a minimum of 3 ${\dot{{\rm{M}}}{\rm{O}}}_{2}$ measurements showed a transition from an oxy-regulating to oxy-conforming state for each fish or fish showed a large drop in ${\dot{{\rm{M}}}{\rm{O}}}_{2}$ and signs of distress in the respirometer. No fish exhibited LoE during trials. Following completion of O_2crit_ trials experimental tanks were aerated with ambient air to swiftly restore O_2_ and CO_2_ levels.

Sea bass were left to recover in respirometers, for a minimum of 1 hour post-trial, until O_2_ levels reached ~21 kPa O_2_ (~100% air saturation). Fish were then removed from respirometers and background respiration was measured for a minimum of 1 hour (6 measurement cycles) for all respirometers immediately post trial.

#### Oxygen consumption rate (${\dot{{\rm{M}}}{\rm{O}}}_{2}$) analysis

Following each 240 s measurement period ${\dot{{\rm{M}}}{\rm{O}}}_{2}$ was automatically calculated by the AquaResp3 software. A linear regression was fitted to the O_2_ versus time data for each measurement period. The slope of this regression (s, kPa O_2_ h^−1^) was then used to calculate ${\dot{{\rm{M}}}{\rm{O}}}_{2}$ (mg O_2_ kg^−1^ h^−1^) using the equation outlined by Svendsen *et al*.^51^:

$$\dot{{\rm{M}}}{{\rm{O}}}_{2}=s{V}_{resp}\,\alpha {m}^{-1}$$

where V_resp_ is the respirometer volume minus the volume of the fish (L), α is the solubility of O_2_ in water (mgO_2_ L^−1^ kPa^−1^) for the relevant salinity and temperature, and m is the mass of the fish (kg). Calculations of ${\dot{{\rm{M}}}{\rm{O}}}_{2}$ where s had a R^2^ of <0.98 were removed from subsequent analysis. For the purpose of establishing O_2crit_ values from a plot of ${\dot{{\rm{M}}}{\rm{O}}}_{2}$ versus ambient O_2_ level, the oxygen saturation of each measurement period was defined as the average of the dissolved O_2_ measurement over the measurement period. The average background respiration, over the 1 hour post-trial measuring period, for each respirometer (average background respiration was <2% of fish ${\dot{{\rm{M}}}{\rm{O}}}_{2}$) was subtracted from ${\dot{{\rm{M}}}{\rm{O}}}_{2}$ measurements to correct fish ${\dot{{\rm{M}}}{\rm{O}}}_{2}$ for background respiration.

We calculated the standard metabolic rate (SMR) in R v3.5.3^54^ using function calcSMR in package fish ${\dot{{\rm{M}}}{\rm{O}}}_{2}$^24^. All ${\dot{{\rm{M}}}{\rm{O}}}_{2}$ values from the overnight recovery period and beginning of the O_2crit_ trial in which average dissolved O_2_ saturation was >80% air saturation were included for SMR calculations. This resulted in approximately 14–16 hours of ${\dot{{\rm{M}}}{\rm{O}}}_{2}$ data to calculate SMR from for each fish. We estimated SMR for all fish using the mean of the lowest 10 ${\dot{{\rm{M}}}{\rm{O}}}_{2}$ measurements during the overnight period. Although Chabot *et al*.^24^ recommend use of the mean of the lowest normal distribution (MLND) or quantile (q = 0.2) methods to calculate SMR we chose the mean of the 10 lowest values as it produced a value of SMR that most accurately matched the consistent low ${\dot{{\rm{M}}}{\rm{O}}}_{2}$ measurements of oxy-regulating fish at values of *p*O_2_ above the O_2crit__point. Additionally, for each fish the coefficient of variation in the mean lowest normal distribution (MLND) was assessed using a ROUT test (Q = 1%) to check whether variation in low ${\dot{{\rm{M}}}{\rm{O}}}_{2}$ measurements was consistent between fish (to account for potential differences in activity in the respirometer prior to O_2crit_ trials). The ROUT test removed one fish from the rising CO_2_ treatment which displayed a CoV of 34.6. Mean CoV of the remaining 15 fish was 11.85 ± 1.26 (±S.E.M).

We then used function calcO2crit from the package to calculate the O_2crit_ for each individual fish, using the estimated SMR of each individual, as detailed in the supplementary material of Claireaux & Chabot^55^. This function identifies the portion of the O_2crit_ test where metabolic rate data follows an oxygen conforming relationship and fits a linear regression line through this data, O_2crit_ is then calculated as the oxygen level at which this regression line crosses the calculated SMR of the individual fish. Plots of ${\dot{{\rm{M}}}{\rm{O}}}_{2}$ against O_2_ during O_2crit_ trials showing calculated O_2crit_ for each individual can be seen in the supplementary material (Supplementary Material Figs 1–16). Calculations were conducted using a gap limit of 0.83 kPa O_2_ (4% air saturation) and a maximum number of 7 ${\dot{{\rm{M}}}{\rm{O}}}_{2}$ points to fit the regression line through the oxygen conforming component of the data used to estimate O_2crit_.

### Measuring blood chemistry

Following O_2crit_ trials sea bass were moved to individual 7 L chambers, which were aerated and supplied with seawater from the aquarium re-circulating system at a rate of about 4 L min^−1^. After an overnight acclimation period we then exposed the fish to a decrease in O_2_ levels to ~6.4 kPa O_2_ (30% air saturation) over a period of 4 hours (equivalent to the rate of decrease in O_2_ used in the previous O_2crit_ tests). We chose to blood sample fish at an O_2_ level above O_2crit_ to ensure that anaerobic metabolism did not influence blood chemistry. This was combined with the same CO_2_ regime each fish experienced during the O_2crit_ test (i.e. either constant or reciprocally rising CO_2_). Once an O_2_ level of ~6.4 kPa O_2_ (30% air saturation) was achieved the fish were allowed to acclimate for 1 hour before being individually anaesthetised *in situ* using a dose of 100 mgL^−1^ of benzocaine. Once fish were judged to be sufficiently anaesthetised (cessation of gill ventilation and lack of response to a pinch of the anal fin) they were immediately transferred within 5 seconds to a gill irrigation table (with the same *p*O_2_ and *p*CO_2_ levels of the respective treatment), where anaesthesia was maintained with a dose of 37.5 mgL^−1^ of benzocaine. Gill ventilation was artificially maintained by a micro-pump, so that the operculum were just open and exhalant water flow could just be visualised. Once a stable gill water flow was established blood was sampled by caudal vessel puncture using a 1 ml heparinised syringe. This method has been demonstrated to obtain accurate measurements of blood chemistry parameters comparable to those achieved using cannulation (Davison & Wilson, University of Exeter, personal communication). At the time of blood sampling water *p*CO_2_ was 656 ± 44 μatm (mean ± S.E.) for fish in the constant ambient CO_2_ regime and 1763 ± 43 μatm (mean ± S.E.) for fish in the progressively rising CO_2_ regime. Water *p*O_2_ was 8.1 ± 0.2 kPa (mean ± S.E., ~38% air saturation) for fish in the constant ambient CO_2_ regime and 8.7 ± 0.2 kPa (mean ± S.E., ~41% air saturation) for fish in the progressively rising CO_2_ regime. Following blood sampling fish were transferred to seawater isolation tanks containing ~20.8 kPa O_2_ (100% air-saturated) to recover from the anaesthetic. They were then monitored over a period of 24 hours before we returned them to their original holding tanks.

Immediately after sampling, whole blood *p*O2 was measured at 18 °C in a temperature-controlled system (Strathkelvin 1302 electrode and 781 meter; Strathkelvin Instruments Ltd, Glasgow, UK). We measured extracellular pH on 30 µL of whole blood using an Accumet Micro pH electrode and Hanna HI8314 pH meter at 18 °C calibrated to pH_NBS_ 7.04 and 9.21 specific buffers. Three 75 µL micro capillary tubes were then filled with whole blood and sealed with Critoseal capillary tube sealant (Fisher) and paraffin oil and centrifuged for 2 minutes at 10,000 rpm. Haematocrit was measured using a Hawksley micro-haematocrit reader. Plasma was extracted from these tubes for analysis of TCO_2_ using a Mettler Toledo 965D carbon dioxide analyser. Plasma *p*CO_2_ and HCO_3_^−^ were then calculated from TCO_2_, temperature and blood pH using the Henderson-Hasselbalch equation with values for solubility and pK^1^_app_ based on Boutilier *et al*.^56,57^. Haemoglobin content of the blood was also assessed by the cyanmethemoglobin method (using Drabkin’s reagent, Sigma). Half the remaining whole blood was then centrifuged at 10,000 rpm for 2 minutes at 4 °C. The resulting plasma was separated and snap frozen in liquid nitrogen and stored at −80 °C before later being used to measure plasma glucose and lactate using a YSI 2900D Biochemistry Analyzer (Xylem Analytics, UK). All measurements or storage of blood occurred within 10 minutes of blood sampling.

### Measuring Hb-oxygen binding

We measured Hb-O_2_ affinity using a Blood Oxygen Binding System (BOBS, Loligo systems), detailed in Oellermann *et al*.^58^. A sample of the same whole blood used for blood chemistry measurements was diluted at a ratio of 1:4 in its own plasma. 1 μL of this blood was then used for measurements. The BOBS exposed this blood sample to gas mixes with a progressive increase in O_2_ whilst measuring absorbance of light across a spectrum ranging from 200 to 800 nm. For each individual fish the gas mix that blood was exposed to matched the calculated *p*CO_2_ of the blood sample. The change in absorption of light at a wavelength of 435 nm was used to assess changes in oxygenation of Hb, as previously used by Verhille & Farrell^59^. Background changes in absorption of the blood sample were corrected using the isosbestic wavelength of 390 nm^59^. Following measurements the BOBS calculated the oxygen equilibrium curve of the sample using Hill’s formula before estimating P_50_^58^.

### Statistical analysis

We conducted all statistical analysis in R v3.5.3^54^. There was no significant difference in mass of fish between the treatment groups (One-Way ANOVA, F_1,13_ = 2.821, p = 0.117) or of estimated SMR of fish between treatment groups (Unpaired t-test, t = 0.8455, d.f. = 13, p = 0.413). There was, however, a significant correlation between estimated SMR and calculated O_2crit_ for all fish (Pearson’s correlation, df = 13, t = 3.32, R = 0.68, p = 0.0056). As such the effect of CO_2_ treatment on O2_crit_ was assessed using an ANCOVA with SMR as a covariate. All other analyses were conducted using general linear modelling (GLM). All values in the text are reported as mean ± standard error (S.E.).

## Supplementary information

Rising CO2 enhances hypoxia tolerance of a marine fish - supplementary materials

## Data Availability

Data is available via the University of Exeter’s online repository at: 10.24378/exe.1523.

## References

[1] Diaz RJ (2001). Overview of Hypoxia around the World. J. Environ. Qual..

[2] Breitburg, D. *et al*. Declining oxygen in the global ocean and coastal waters. *Science* (*80-*). **359** (2018). 10.1126/science.aam7240 29301986

[3] Diaz RJ, Rosenberg R (2008). Spreading Dead Zones and Consequences for Marine Ecosystems. Science (80-)..

[4] Townhill BL (2017). Consequences of climate-induced low oxygen conditions for commercially important fish. Mar. Ecol. Prog. Ser..

[5] Robinson C (2019). Microbial Respiration, the Engine of Ocean Deoxygenation. Frontiers in Marine Science..

[6] Rabalais NN, Turner RE, Díaz RJ, Justić D (2009). Global change and eutrophication of coastal waters. ICES J. Mar. Sci..

[7] Sunda WG, Cai W-J (2012). Eutrophication Induced CO_2_-Acidification of Subsurface Coastal Waters: Interactive Effects of Temperature, Salinity, and Atmospheric PCO_2_. Environ. Sci. Technol..

[8] Wallace RB, Baumann H, Grear JS, Aller RC, Gobler CJ (2014). Coastal ocean acidification: The other eutrophication problem. Estuar. Coast. Shelf Sci..

[9] Gobler, C. J. & Baumann, H. Hypoxia and acidification in ocean ecosystems: coupled dynamics and effects on marine life. *Biol*. *Lett*. **12** (2016). 10.1098/rsbl.2015.0976 PMC4892234 27146441

[10] Altieri AH, Gedan KB (2015). Climate change and dead zones. Glob. Chang. Biol..

[11] Oschlies A, Brandt P, Stramma L, Schmidtko S (2018). Drivers and mechanisms of ocean deoxygenation. Nat. Geosci..

[12] Oschlies, A., Schulz, K. G., Riebesell, U. & Schmittner, A. Simulated 21^st^ century’s increase in oceanic suboxia by CO_2_-enhanced biotic carbon export. *Global Biogeochem*. *Cycles* **22** (2008).

[13] Doney SC, Fabry VJ, Feely RA, Kleypas JA (2009). Ocean Acidification: The Other CO_2_ Problem. Ann. Rev. Mar. Sci..

[14] Melzner F (2013). Future ocean acidification will be amplified by hypoxia in coastal habitats. Mar. Biol..

[15] Brandt SB, Gerken M, Hartman KJ, Demers E (2009). Effects of hypoxia on food consumption and growth of juvenile striped bass (Morone saxatilis). J. Exp. Mar. Bio. Ecol..

[16] Almeida LZ, Guffey SC, Sepúlveda MS, Höök TO (2017). Behavioral and physiological responses of yellow perch (Perca flavescens) to moderate hypoxia. Comp. Biochem. Physiol. Part A Mol. Integr. Physiol..

[17] Chabot D, Dutil JD (1999). Reduced growth of Atlantic cod in non-lethal hypoxic conditions. J. Fish Biol..

[18] Domenici P, Steffensen JF, Batty RS (2000). The effect of progressive hypoxia on swimming activity and schooling in Atlantic herring. J. Fish Biol..

[19] Gobler CJ, DePasquale EL, Griffith AW, Baumann H (2014). Hypoxia and Acidification Have Additive and Synergistic Negative Effects on the Growth, Survival, and Metamorphosis of Early Life Stage Bivalves. PLoS One.

[20] Steckbauer A (2015). Synergistic effects of hypoxia and increasing CO_2_ on benthic invertebrates of the central Chilean coast. Front. Mar. Sci..

[21] Miller SH, Breitburg DL, Burrell RB, Keppel AG (2016). Acidification increases sensitivity to hypoxia in important forage fishes. Mar. Ecol. Prog. Ser..

[22] Nikinmaa M (1997). Oxygen and carbon dioxide transport in vertebrate erythrocytes: an evolutionary change in the role of membrane transport. J. Exp. Biol..

[23] Rummer JL, Brauner CJ (2015). Root effect haemoglobins in fish may greatly enhance general oxygen delivery relative to other vertebrates. PLoS One.

[24] Chabot D, Steffensen JF, Farrell AP (2016). The determination of standard metabolic rate in fishes. J. Fish Biol..

[25] Perry, S. F., Jonz, M. G. & Gilmour, K. M. Chapter 5 Oxygen Sensing And The Hypoxic Ventilatory Response. In *Fish Physiology* (eds J. G. Richards, A. P. F. and C. J. B.) Volume 27, 193–253 (Academic Press, 2009).

[26] Wells, R. M. G. Chapter 6 Blood‐Gas Transport and Hemoglobin Function: Adaptations for Functional and Environmental Hypoxia. In *Fish Physiology* (eds Jeffrey G. Richards, A. P. F. and C. J. B.) Volume 27, 255–299 (Academic Press, 2009).

[27] Gamperl, A. K. & Driedzic, W. R. Chapter 7 Cardiovascular Function and Cardiac Metabolism. In *Hypoxia* (eds Richards, J. G., Farrell, A. P. & Brauner, C. J. B. T.-F. P.) **27**, 301–360 (Academic Press, 2009).

[28] Wood CM (2018). The fallacy of the P_crit_ – are there more useful alternatives?. J. Exp. Biol..

[29] Speers-Roesch B, Mandic M, Groom DJE, Richards JG (2013). Critical oxygen tensions as predictors of hypoxia tolerance and tissue metabolic responses during hypoxia exposure in fishes. J. Exp. Mar. Bio. Ecol..

[30] Regan MD (2019). Don’t throw the fish out with the respirometry water. J. Exp. Biol..

[31] Pelster, B. & Weber, R. E. The Physiology of the Root Effect. In *Advances in Comparative and Environmental Physiology* 51–77, 10.1007/978-3-642-75900-0_2LB-Pelster1991 (Springer Berlin Heidelberg, 1991).

[32] Nikinmaa M (1983). Adrenergic regulation of haemoglobin oxygen affinity in rainbow trout red cells. J. Comp. Physiol..

[33] Tetens V, Christensen NJ (1987). Beta-adrenergic control of blood oxygen affinity in acutely hypoxia exposed rainbow trout. J. Comp. Physiol. B.

[34] Weber RE, Lykkeboe G (1978). Respiratory adaptations in carp blood influences of hypoxia, red cell organic phosphates, divalent cations and CO_2_ on hemoglobin-oxygen affinity. J. Comp. Physiol..

[35] Soivio A, Nikinmaa M, Westman K (1980). The blood oxygen binding properties of hypoxicSalmo gairdneri. J. Comp. Physiol..

[36] Mairbäurl, H. & Weber, R. E. Oxygen Transport by Hemoglobin. *Comprehensive Physiology*, 10.1002/cphy.c080113 (2012). 10.1002/cphy.c080113 23798307

[37] Esbaugh AJ, Heuer R, Grosell M (2012). Impacts of ocean acidification on respiratory gas exchange and acid-base balance in a marine teleost, Opsanus beta. J. Comp. Physiol. B..

[38] Perry SF, Goss GG, Laurent P (1992). The interrelationships between gill chloride cell morphology and ionic uptake in four freshwater teleosts. Can. J. Zool..

[39] Rogers, N. J., Urbina, M. A., Reardon, E. E., McKenzie, D. J. & Wilson, R. W. A new analysis of hypoxia tolerance in fishes using a database of critical oxygen level (Pcrit). *Conserv*. *Physiol*. **4** (2016). 10.1093/conphys/cow012 PMC4849809 27293760

[40] Steffensen JF (1989). Some errors in respirometry of aquatic breathers: How to avoid and correct for them. Fish Physiol. Biochem..

[41] Regan MD, Richards JG (2017). Rates of hypoxia induction alter mechanisms of O_2_ tension of goldfish. J. Exp. Biol..

[42] Snyder S (2016). Effect of closed v. intermittent-flow respirometry on hypoxia tolerance in the shiner perch Cymatogaster aggregata. J. Fish Biol..

[43] Hancock JR, Place SP (2016). Impact of ocean acidification on the hypoxia tolerance of the woolly sculpin, Clinocottus analis. Conserv. Physiol..

[44] Cochran RE, Burnett LE (1996). Respiratory responses of the salt marsh animals, Fundulus heteroclitus, Leiostomus xanthurus, and Palaemonetes pugio to environmental hypoxia and hypercapnia and to the organophosphate pesticide, azinphosmethyl. J. Exp. Mar. Bio. Ecol..

[45] Kramer DL, McClure M (1982). Aquatic surface respiration, a widespread adaptation to hypoxia in tropical freshwater fishes. Environ. Biol. Fishes.

[46] Dixon RL, Grecay PA, Targett TE (2017). Responses of juvenile Atlantic silverside, striped killifish, mummichog, and striped bass to acute hypoxia and acidification: Aquatic surface respiration and survival. J. Exp. Mar. Bio. Ecol..

[47] DePasquale E, Baumann H, Gobler CJ (2015). Vulnerability of early life stage Northwest Atlantic forage fish to ocean acidification and low oxygen. Mar. Ecol. Prog. Ser..

[48] Komoroske, L. M. *et al*. Ontogeny influences sensitivity to climate change stressors in an endangered fish. *Conserv*. *Physiol*. **2** (2014). 10.1093/conphys/cou008 PMC4806739 27293629

[49] Murray CS, Malvezzi A, Gobler CJ, Baumann H (2014). Offspring sensitivity to ocean acidification changes seasonally in a coastal marine fish. Mar. Ecol. Prog. Ser..

[50] Baumann, H. Experimental assessments of marine species sensitivities to ocean acidification and co-stressors: how far have we come? *Can*. *J*. *Zool*. 399–408, 10.1139/cjz-2018-0198 (2019).

[51] Svendsen MBS, Bushnell PG, Steffensen JF (2016). Design and setup of intermittent-flow respirometry system for aquatic organisms. J. Fish Biol..

[52] Del Giorgio PA, Duarte CM (2002). Respiration in the open ocean. Nature.

[53] Lewis C, Clemow K, Holt WV (2013). Metal contamination increases the sensitivity of larvae but not gametes to ocean acidification in the polychaete Pomatoceros lamarckii (Quatrefages). Mar. Biol..

[54] R Core Team. R: A language and environment for statistical computing (2019).

[55] Claireaux G, Chabot D (2016). Responses by fishes to environmental hypoxia: integration through Fry’s concept of aerobic metabolic scope. J. Fish Biol..

[56] Boutilier RG, Heming TA, Iwama GK (1984). Appendix-physicochemical parameters for use in fish respiratory physiology. Fish Physiol..

[57] Boutilier RG, Iwama GK, Heming TA, Randall DJ (1985). The apparent pK of carbonic acid in rainbow trout blood plasma between 5 and 15°C. Respir. Physiol..

[58] Oellermann M, Pörtner H-O, Mark FC (2014). Simultaneous high-resolution pH and spectrophotometric recordings of oxygen binding in blood microvolumes. J. Exp. Biol..

[59] Verhille C, Farrell AP (2012). The *in vitro* blood-O_2_ affinity of triploid rainbow trout Oncorhynchus mykiss at different temperatures and CO_2_ tensions. J. Fish Biol..

