# Supplementary material for: Rising CO2 enhances hypoxia tolerance in a marine fish
Source: Sci Rep. 2019 Oct 22;9:15152. doi: 10.1038/s41598-019-51572-4 (PMC6805886; doi:10.1038/s41598-019-51572-4)
Supplement: Supplementary file 1 — Rising CO2 enhances hypoxia tolerance of a marine fish - supplementary materials [file 41598_2019_51572_MOESM1_ESM.pdf]

Rising CO_2 enhances hypoxia tolerance in a marine fish

Daniel W. Montgomery[a*], Stephen D. Simpson[a], Georg H. Engelhard[bc], Silvana N.R. Birchenough[b], Rod W. Wilson[a*]

[*]co-corresponding authors – dm513@exeter.ac.uk, R.W.Wilson@exeter.ac.uk

[a] Bioscience Department, College of Life and Environmental Sciences, University of Exeter, UK

[b] Centre for Environment, Fisheries & Aquaculture Science (Cefas), Pakefield Road, Lowestoft NR33 0HT, UK

[c] School of Environmental Sciences, University of East Anglia, Norwich NR4 7TJ, UK

Supplementary material

Table 1: Calculated pCO_2 during decline in O_2 (kPa) during a simulated hypoxic event at 18°C and a salinity of 35. Calculations were performed in the widely used program CO2SYS v2.1 using K1, K2 constants from Mehrbach et al. 1973 refitted by Dickson and Molero, 1987, $KHSO_4$ values from Dickson, the NBS pH scale and $[B]_s$ values from Uppstrom, 1974. Increase in Total CO_2 (or total DIC) during hypoxic event was calculated assuming a respiratory quotient of 1:1 for bacteria based on Del Giorgio and Duarte, 2002.

	pCO₂ (µatm)
Oxygen (kPa)	18°C
20.79	400
16.63	541
12.47	758
8.31	1105
6.24	1350
4.16	1654
3.12	1831

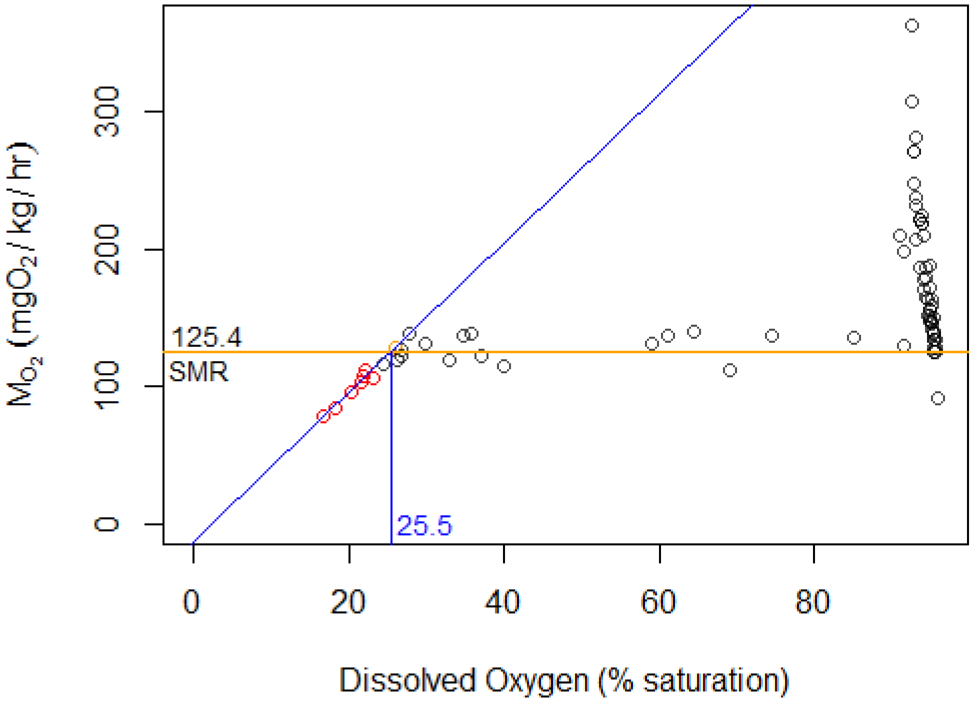

Figure 1: $\dot{M}O_2$ profile for fish #1 exposed to decreasing O_2 with a constant CO_2. The plot shows calculated SMR as well as the linear regression calculated in R using package fishMO2 when $\dot{M}O_2$ is judged to show an oxy-conforming relationship. O_{2crit} is determined as the point the linear regression and SMR line meet.

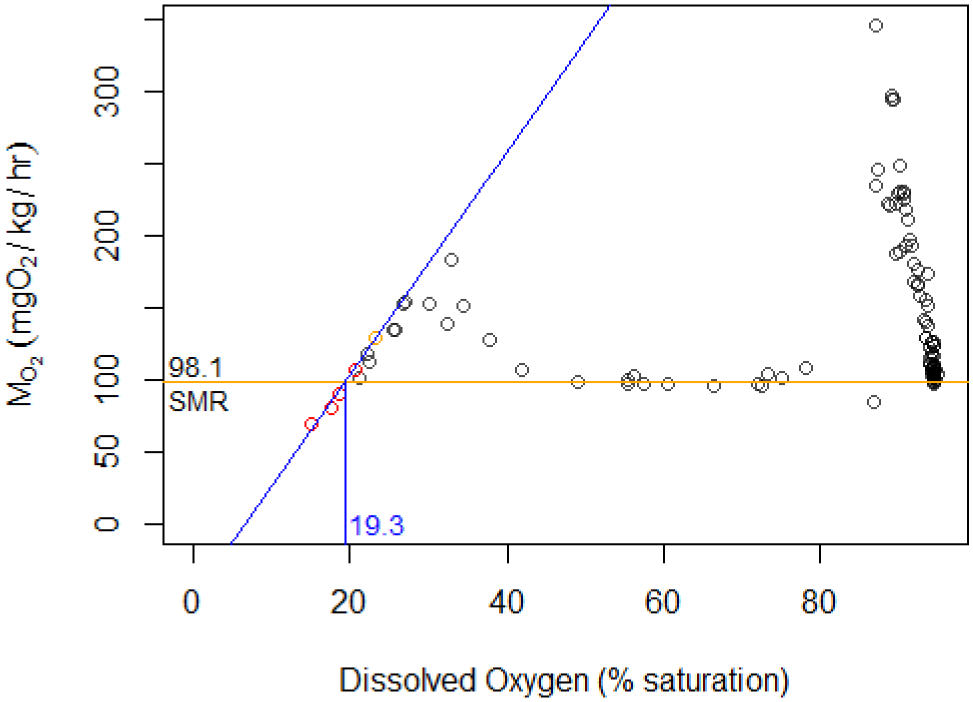

Figure 2: $\dot{M}O_2$ profile for fish #2 exposed to decreasing O_2 with a constant CO_2. The plot shows calculated SMR as well as the linear regression calculated in R using package fishMO2 when $\dot{M}O_2$ is judged to show an oxy-conforming relationship. O_{2crit} is determined as the point the linear regression and SMR line meet.

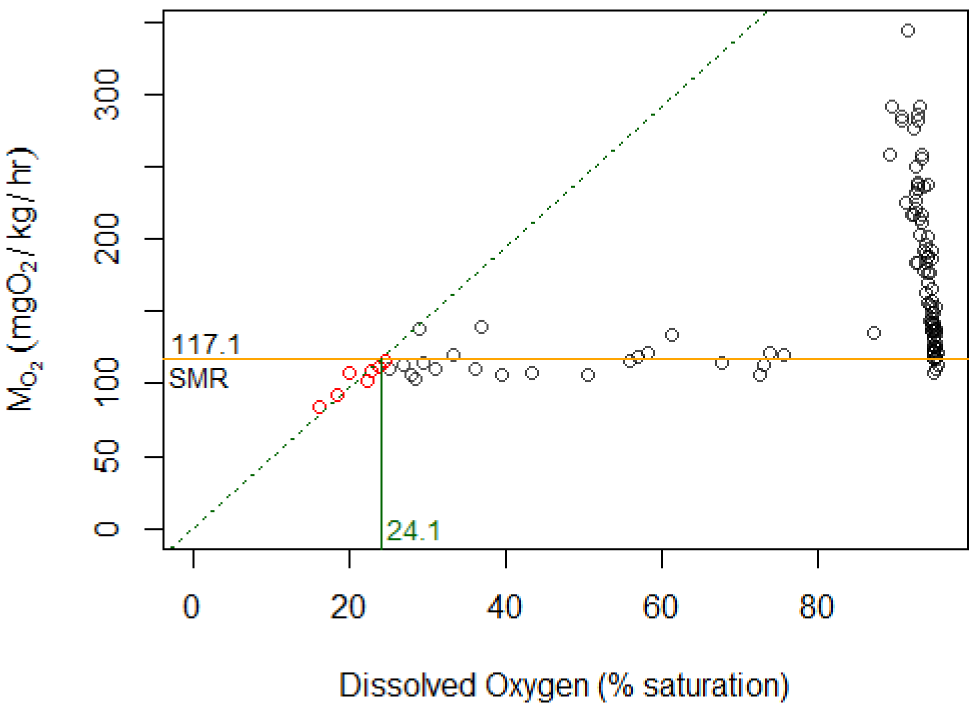

Figure 3: $\dot{M}O_2$ profile for fish #3 exposed to decreasing O_2 with a constant CO_2. The plot shows calculated SMR as well as the linear regression calculated in R using package fishMO2 when $\dot{M}O_2$ is judged to show an oxy-conforming relationship. O_{2crit} is determined as the point the linear regression and SMR line meet.

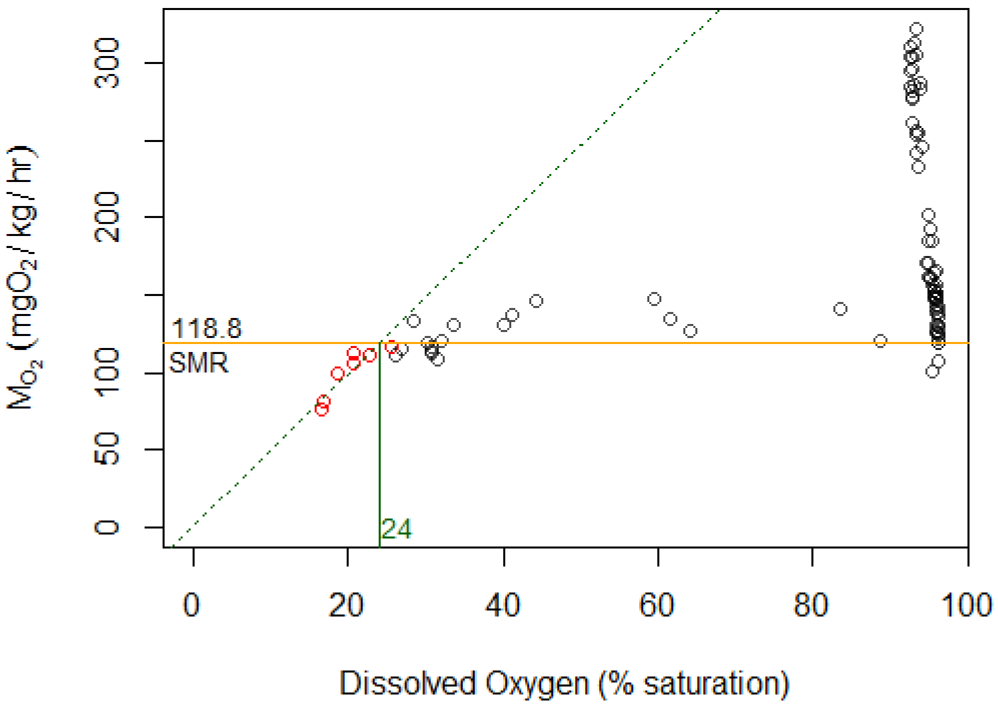

Figure 4: $\dot{M}O_2$ profile for fish #4 exposed to decreasing O_2 with a constant CO_2. The plot shows calculated SMR as well as the linear regression calculated in R using package fishMO2 when $\dot{M}O_2$ is judged to show an oxy-conforming relationship. O_{2crit} is determined as the point the linear regression and SMR line meet.

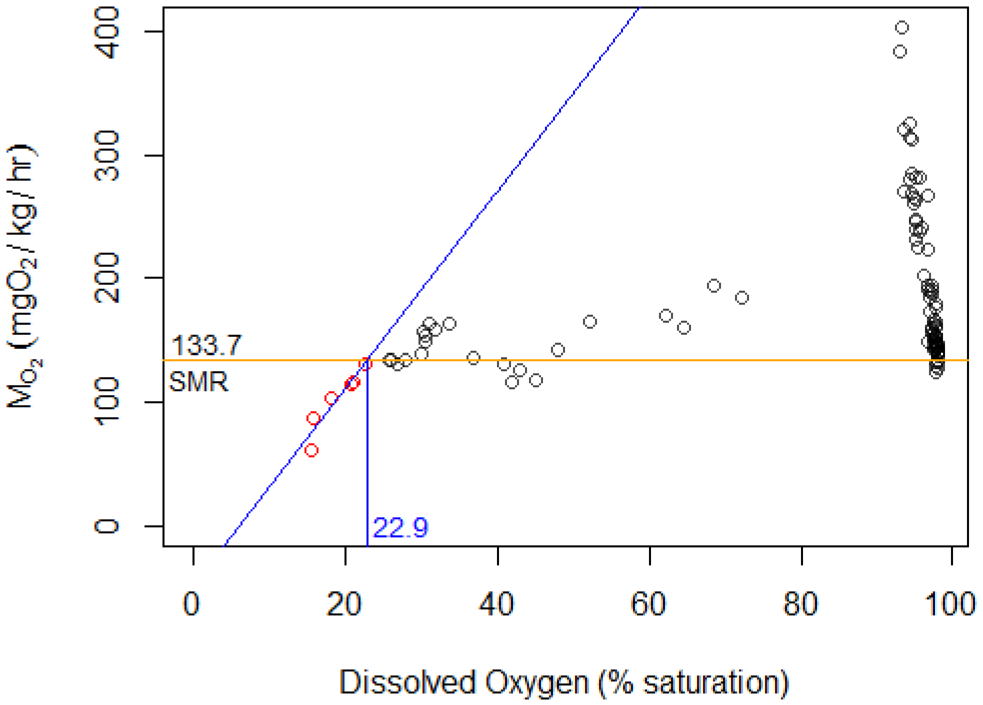

Figure 5: $\dot{M}O_2$ profile for fish #5 exposed to decreasing O_2 with a constant CO_2. The plot shows calculated SMR as well as the linear regression calculated in R using package fishMO2 when $\dot{M}O_2$ is judged to show an oxy-conforming relationship. O_{2crit} is determined as the point the linear regression and SMR line meet.

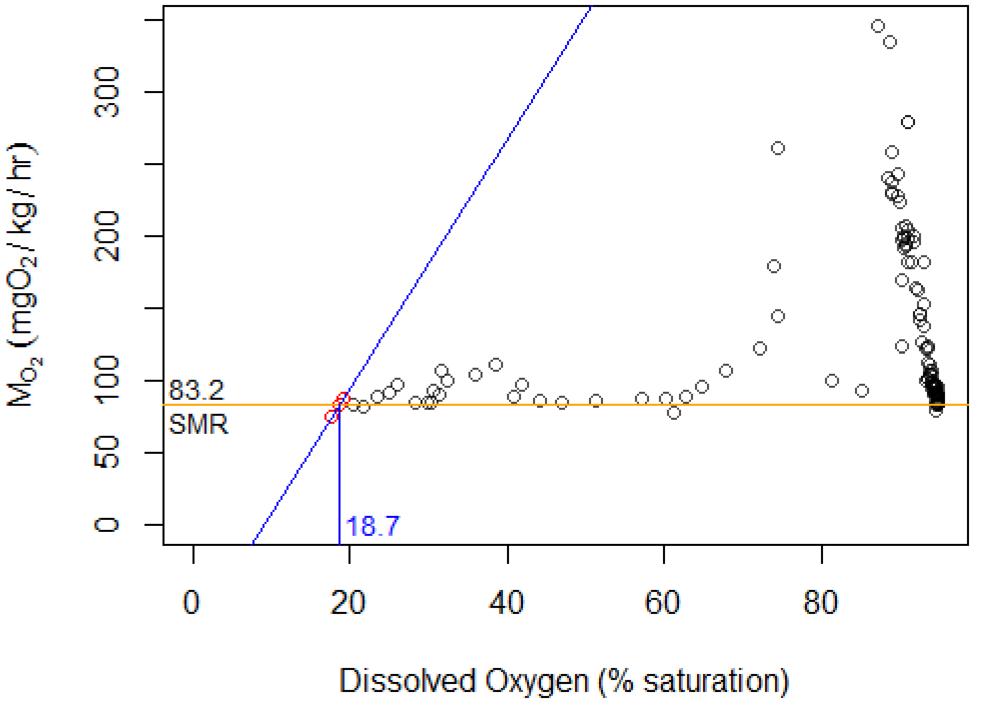

Figure 6: $\dot{M}O_2$ profile for fish #6 exposed to decreasing O_2 with a constant CO_2. The plot shows calculated SMR as well as the linear regression calculated in R using package fishMO2 when $\dot{M}O_2$ is judged to show an oxy-conforming relationship. O_{2crit} is determined as the point the linear regression and SMR line meet.

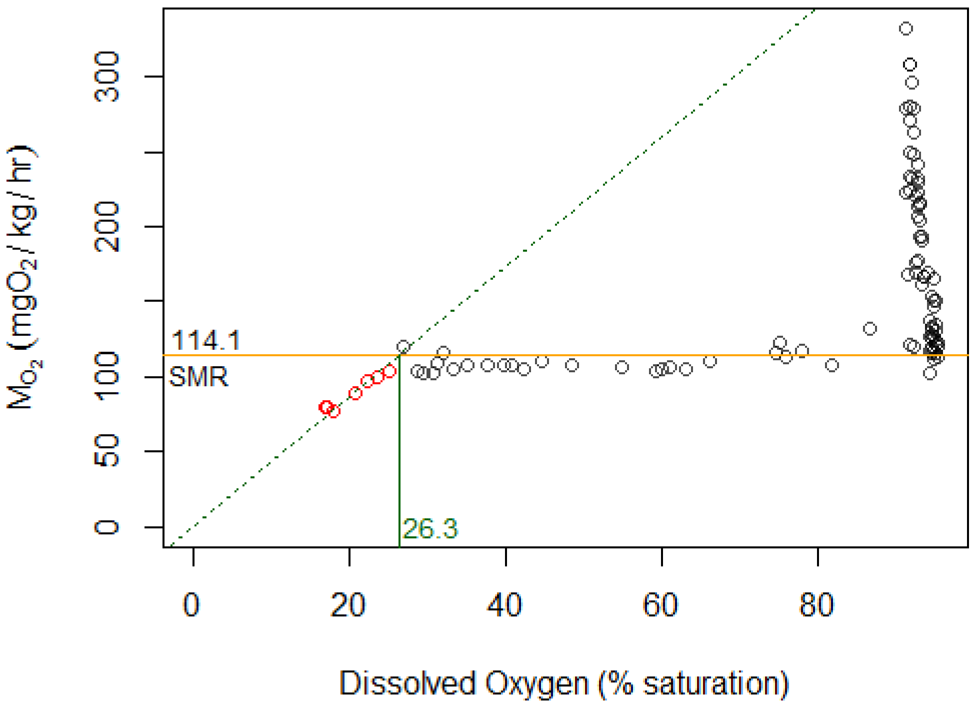

Figure 7: $\dot{M}O_2$ profile for fish #7 exposed to decreasing O_2 with a constant CO_2. The plot shows calculated SMR as well as the linear regression calculated in R using package fishMO2 when $\dot{M}O_2$ is judged to show an oxy-conforming relationship. O_{2crit} is determined as the point the linear regression and SMR line meet.

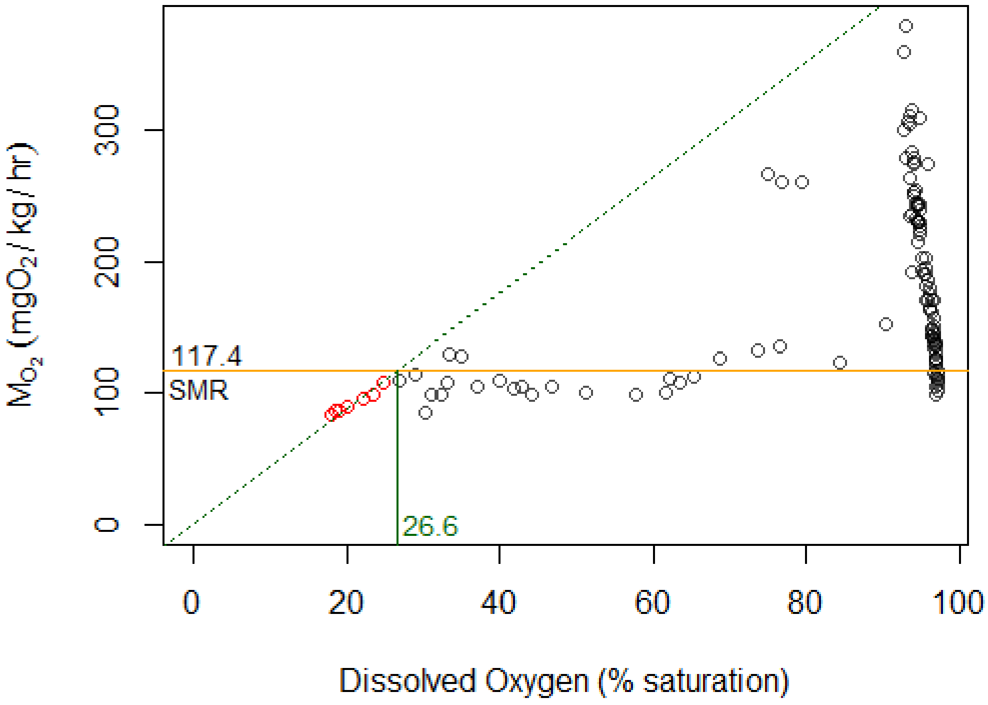

Figure 8: $\dot{M}O_2$ profile for fish #8 exposed to decreasing O_2 with a constant CO_2. The plot shows calculated SMR as well as the linear regression calculated in R using package fishMO2 when $\dot{M}O_2$ is judged to show an oxy-conforming relationship. O_{2crit} is determined as the point the linear regression and SMR line meet.

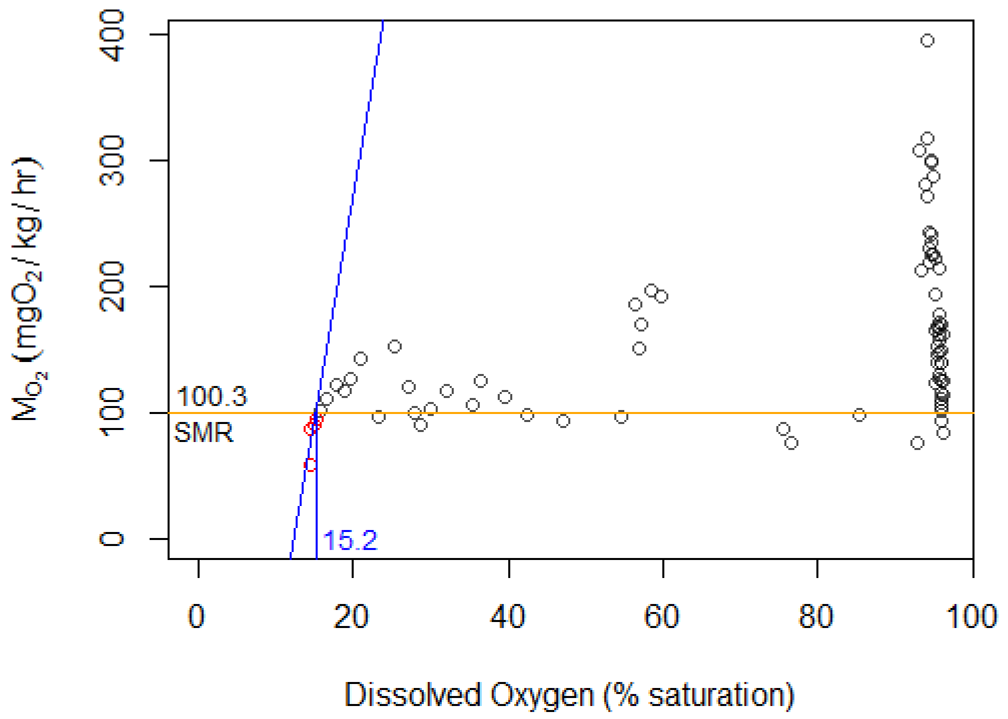

Figure 9: $\dot{M}O_2$ profile for fish #9 exposed to decreasing O_2 with rising CO_2. The plot shows calculated SMR as well as the linear regression calculated in R using package fishMO2 when $\dot{M}O_2$ is judged to show an oxy-conforming relationship. O_{2crit} is determined as the point the linear regression and SMR line meet.

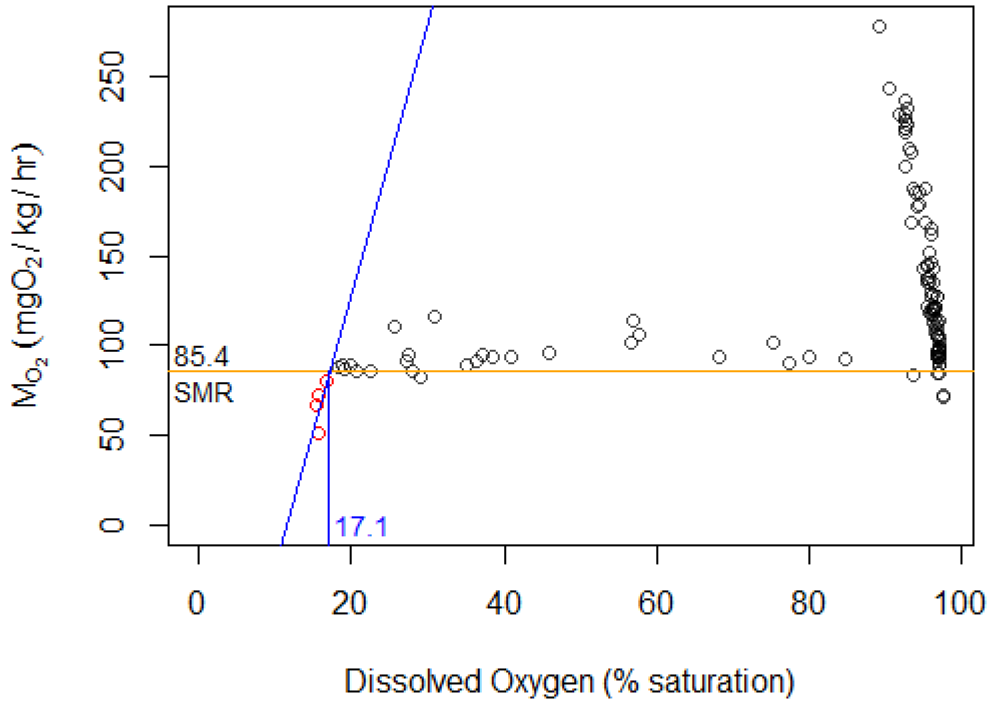

Figure 10: $\dot{M}O_2$ profile for fish #10 exposed to decreasing O_2 with rising CO_2. The plot shows calculated SMR as well as the linear regression calculated in R using package fishMO2 when $\dot{M}O_2$ is judged to show an oxy-conforming relationship. O_{2crit} is determined as the point the linear regression and SMR line meet.

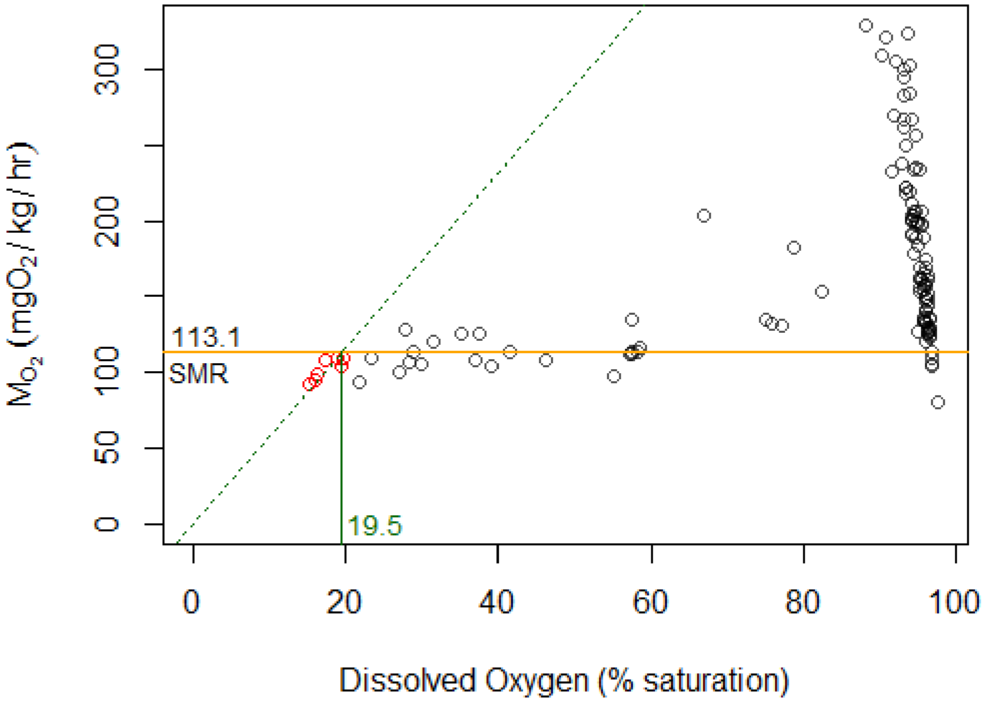

Figure 11: $\dot{M}O_2$ profile for fish #11 exposed to decreasing O_2 with rising CO_2. The plot shows calculated SMR as well as the linear regression calculated in R using package fishMO2 when $\dot{M}O_2$ is judged to show an oxy-conforming relationship. O_{2crit} is determined as the point the linear regression and SMR line meet.

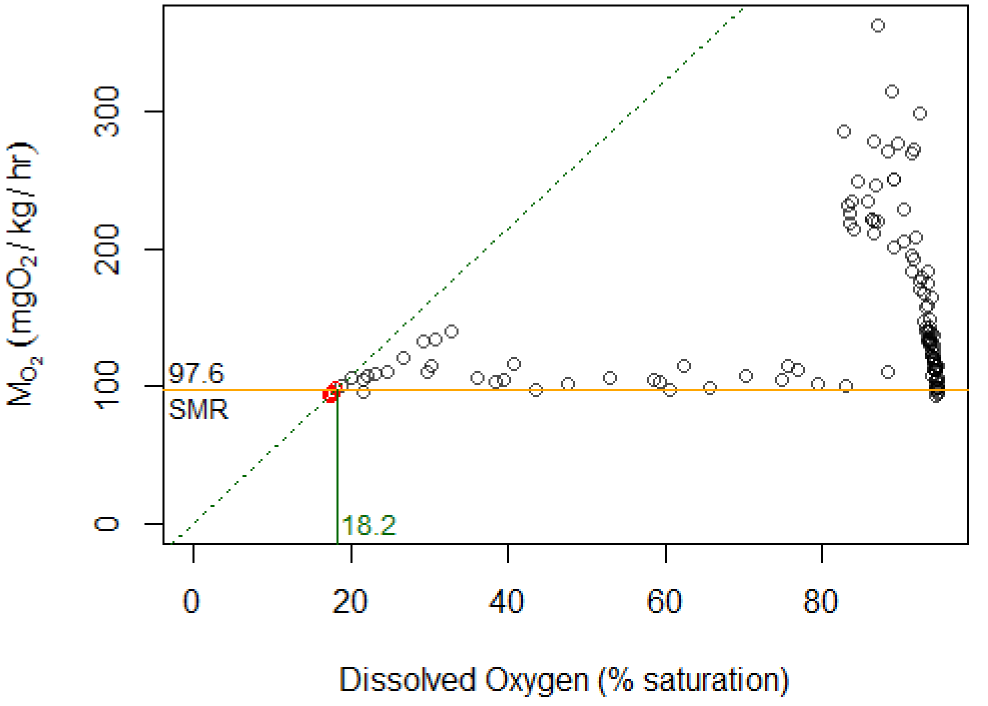

Figure 12: $\dot{M}O_2$ profile for fish #12 exposed to decreasing O_2 with rising CO_2. The plot shows calculated SMR as well as the linear regression calculated in R using package fishMO2 when $\dot{M}O_2$ is judged to show an oxy-conforming relationship. O_{2crit} is determined as the point the linear regression and SMR line meet.

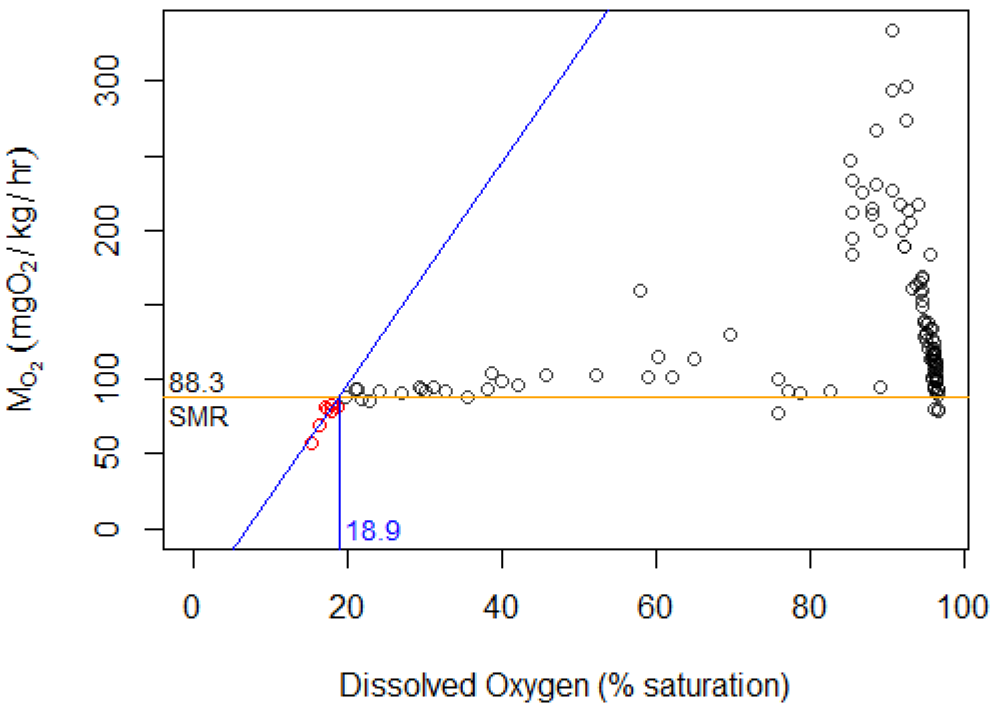

Figure 13: $\dot{M}O_2$ profile for fish #13 exposed to decreasing O_2 with rising CO_2. The plot shows calculated SMR as well as the linear regression calculated in R using package fishMO2 when $\dot{M}O_2$ is judged to show an oxy-conforming relationship. O_{2crit} is determined as the point the linear regression and SMR line meet.

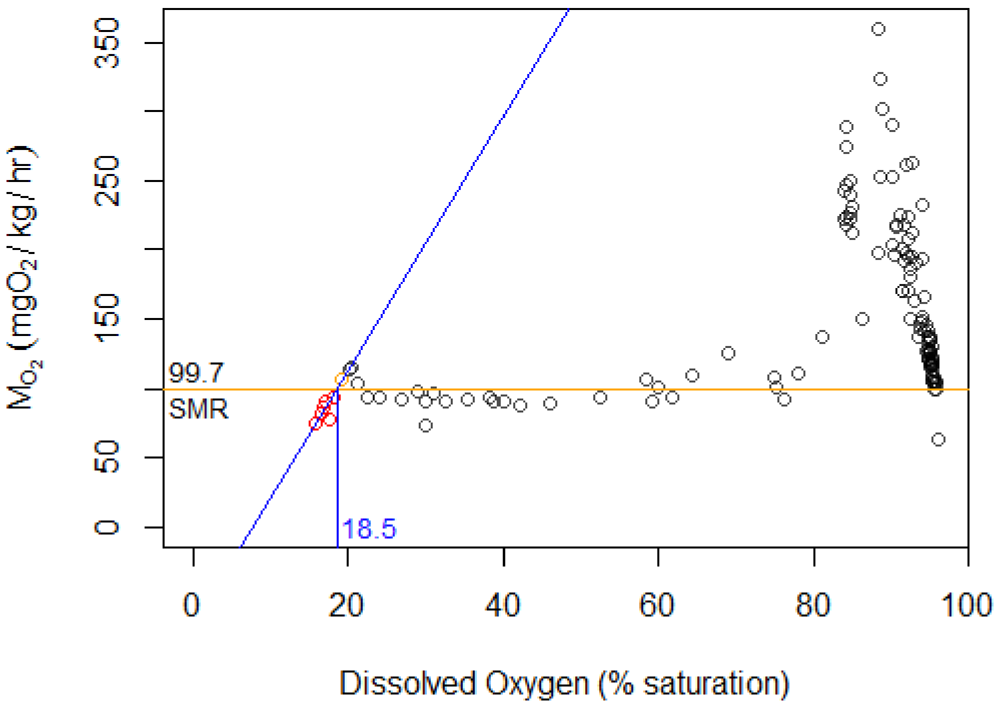

Figure 14: $\dot{M}O_2$ profile for fish #14 exposed to decreasing O_2 with rising CO_2. The plot shows calculated SMR as well as the linear regression calculated in R using package fishMO2 when $\dot{M}O_2$ is judged to show an oxy-conforming relationship. O_{2crit} is determined as the point the linear regression and SMR line meet.

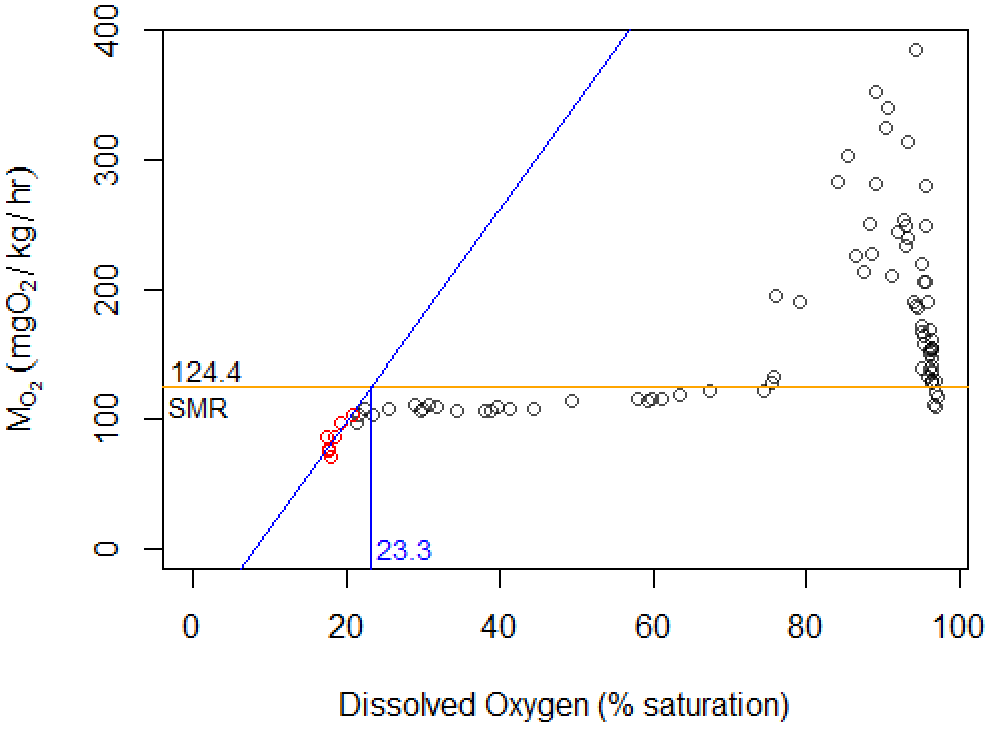

Figure 15: $\dot{M}O_2$ profile for fish #15 exposed to decreasing O_2 with rising CO_2. The plot shows calculated SMR as well as the linear regression calculated in R using package fishMO2 when $\dot{M}O_2$ is judged to show an oxy-conforming relationship. O_{2crit} is determined as the point the linear regression and SMR line meet.

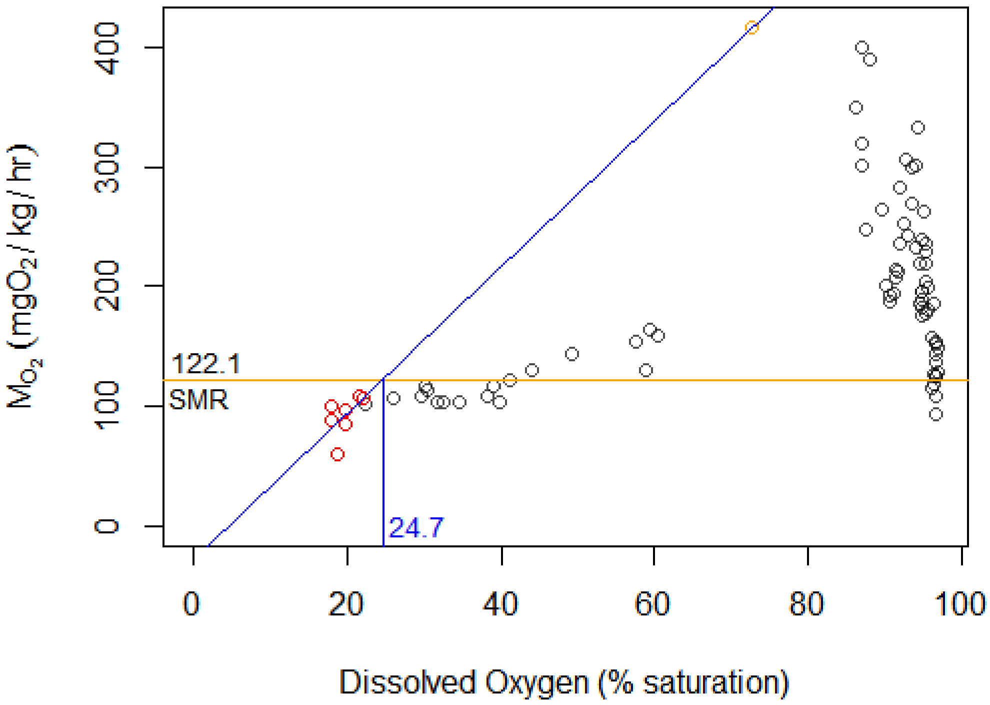

Figure 16: $\dot{M}O_2$ profile for fish #16 exposed to decreasing O_2 with rising CO_2. The plot shows calculated SMR as well as the linear regression calculated in R using package fishMO2 when $\dot{M}O_2$ is judged to show an oxy-conforming relationship. O_{2crit} is determined as the point the linear regression and SMR line meet. SMR and O_{2crit} data from fish #16 wasn't included in subsequent analysis as the fish did not acclimate well to the respirometer leading to an overestimation of SMR.
